# Effect of family socio-economic status on subjective well-being among Norwegian adolescents: Mediation and moderation effects by general self-efficacy from a gendered perspective

**DOI:** 10.1186/s12889-025-24697-7

**Published:** 2025-10-08

**Authors:** Catherine Anne Nicole Lorentzen, Lars Bauger, Eliva Atieno Ambugo

**Affiliations:** Department of Health, Social, and Welfare Studies, University of South-Eastern Norway, Post Office Box 4, 3199 Borre, Norway

**Keywords:** Adolescents, Family socio-economic status, Subjective well-being, General self-efficacy, Mediation, Moderation, Gender perspective

## Abstract

**Background:**

In order to better target public health interventions addressing socio-economic disparities in mental health among adolescents, a better understanding of this phenomenon is needed. This study aimed at investigating the mediating and moderating roles of adolescents´ general self-efficacy in the relationship between their family socio-economic status and their subjective well-being, and the dependence of these effects on gender.

**Methods:**

The study was based on data from the 2021 cross-sectional Ungdata survey among 17,941 adolescents aged 14 to 19 in a Norwegian county. Four multivariable linear regression-based mediation/moderation analyses were conducted: a simple mediation model tested whether the effect of family socio-economic status on well-being was mediated by general self-efficacy, a moderated mediation model tested whether such a mediation effect was moderated by gender, a simple moderation model tested whether general self-efficacy moderated the effect of family socio-economic status on well-being, and a moderated moderation model tested whether such an interaction effect was moderated by gender.

**Results:**

We found that some of the positive effect of the adolescents´ family socio-economic status on their well-being was mediated by their general self-efficacy beliefs. This indirect effect was stronger for girls than for boys due to the stronger positive association between general self-efficacy and well-being among girls. General self-efficacy also moderated the association between family socio-economic status and well-being, i.e. general self-efficacy slightly protected against reduced well-being among adolescents living in families with fewer socio-economic resources. This moderation effect was not dependent on gender.

**Conclusions:**

These findings enhance our understanding of the pathways by which socio-economic status affects adolescent mental health and the factors that may protect against the negative influences of living in socio-economically less advantaged circumstances. From a public mental health perspective with particular focus on reducing inequalities in well-being among adolescents, it appears especially fruitful to support the strengthening of general self-efficacy beliefs among girls and among adolescents from lower socio-economic households.

**Supplementary Information:**

The online version contains supplementary material available at 10.1186/s12889-025-24697-7.

## Background

Numerous studies from Western societies, including Norway, show that adolescents´ reported mental health has worsened over the past decade [1–4]. This relates both to the negative dimension of mental health, i.e. symptoms of mental illness, and the positive dimension, i.e. perceptions of well-being [5, 6]. This development highlights the need to further explore, identify, and understand influencing factors that may be addressed in public health efforts to alleviate this trend. In addition to factors such as academic pressure, smart phone use, and earlier onset of puberty that are thought to contribute to this trend [7, 8], many international and national studies point to the socio-economic situation of the family as a major factor impacting children`s and adolescents` present and future mental health [2, 9–11]. These studies typically reveal a gradient-like positive association whereby higher socio-economic status (SES) is associated with better mental health. Various explanations for this social gradient in mental health have been proposed, including uneven distribution of material, behavioral, social, psychological, and biological resources pertinent to mental health between different SES groups [9]. It appears that observed health inequalities have persisted over time despite large public health efforts to reduce them through welfare policies and structural measures [2, 9, 12]. In order to better target public health interventions aimed at addressing socio-economic disparities in mental health among adolescents, the literature calls for a better understanding of this phenomenon with regard to: the pathways through which SES affects mental health [13, 14], factors that may protect against the negative influence of living in a family with fewer socio-economic resources [13, 15, 16], and whether such mediating and moderating effects differ across subgroups of the adolescent population [15].

A psychological factor that has received little attention as a possible pathway through which SES affects mental health is general self-efficacy. General self-efficacy refers to a broad and stable sense of being able to cope with stressful situations and challenges in life [17, 18]. A growing number of cross-sectional and longitudinal studies report positive associations between adolescents` general self-efficacious perceptions and negative (e.g., [11, 19]) and positive (e.g., [20–24]) mental health outcomes. This relationship can be explained through several pathways. Firstly, having optimistic beliefs about one´s own competence and ability to tackle upcoming demanding situations is likely to result in more positive affect [25]. Second, people with higher general self-efficacy beliefs are more inclined to perceive difficult tasks and circumstances as opportunities for mastery, to set more ambitious goals, and to be persistent in pursuing those goals. This stronger engagement and dedication across different activities is likely to result in greater enjoyment and satisfaction with life [17, 18, 26]. Finally, people with higher general self-efficacy will experience more mental health benefits due to the higher adoption of behaviors that promote mental health, such as physical and social activities [25, 27].

Although less studied, some researchers have identified a positive relationship between adolescents` family SES and their general self-efficacy [27, 28]. There are several possible pathways for how living in a family with more socio-economic resources relates to higher general self-efficacy, and they reflect what theory and research highlight as the main sources of self-efficacy. Hierarchically ordered in accordance to their anticipated power of influence, these sources include: 1) direct personal experiences of mastery, when success is attributed internally, as it provides insight into one´s abilities and informs beliefs about the likelihood of future success; 2) vicarious self-efficacy experiences, i.e., when your own self-efficacy beliefs are strengthened by observing social role models master difficult tasks and situations; and 3) social persuasion, which refers to strengthened self-efficacy as a result of significant others verbally convincing you about your capabilities [26, 29, 30]. A wide array of studies demonstrate the effects of SES on outcomes that may influence adolescents´ access to experiences of mastery, successful role models, and encouraging social environments. Firstly, much research has shown that lower family SES is related to poorer parenting practices and home environment through parental psychological distress, less childrearing knowledge, lower expectations regarding achievement, and less available time for the child [15, 31, 32]. This has been found to include a greater likelihood of reduced parental support and engagement, increased disorganization within the home, and more frequent family conflicts. Studies further demonstrate that, to name a few: lower family SES is associated with a smaller family social network [9]; poorer physical home environments [9, 32]; having fewer friends and experiencing more bullying [9, 33]; having a poorer relationship with one´s teachers and other pupils [9, 33]; lower access to good quality and safe facilities for activities in the neighborhood [9, 34]; lower participation in organized leisure activities [9]; poorer neurocognitive functioning, including language, executive function, and memory ability [32, 35, 36], and related poorer academic skills and performance [9]; as well as less favorable lifestyle behaviors [14, 34, 37, 38].

To our knowledge, only two studies, a Danish and a Polish one, have previously investigated and found support for general self-efficacy as a mediator of the association between family SES and mental health among adolescents [28, 39]. Further supporting the aforementioned pathway are studies that have shown general self-efficacy to be a contributing mechanism for the association between positive parenting practices and adolescents´ mental health [40, 41], and studies that have demonstrated inverse associations between several of the other aforementioned outcomes of low family SES (e.g., lower academic achievements, less participation in organized leisure activities) and general self-efficacy among adolescents [42–46]. Based on this brief review of the literature, it is likely that some of the effects of adolescents´ family SES on their mental health occur through their self-efficacy beliefs. Testing this assumption will contribute to the sparse theoretical knowledge regarding the pathways by which SES affects mental health outcomes among adolescents.

In addition to possibly acting as a mediator for the effect of family SES on mental health, it is also of interest to examine whether general self-efficacy moderates such effects. This dual role of general self-efficacy as both a mediator and a moderator for the relationship between family SES and mental health appears possible considering that general self-efficacy seems to be influenced by a number of environmental factors independent of family SES [46, 47]. Since general self-efficacy may be more amenable to change than family socio-economic resources, its potential role as a buffer against negative mental health consequences of living in a less socio-economically advantaged household is particularly important to identify. This proposition is supported by the only study we have identified that has tested this assumption [16]. Among 3,969 11–15 years old Danish schoolchildren, high general self-efficacy was found to modify the association between parental occupational class and mental health, i.e. high general self-efficacy seemed to protect adolescents from lower socio-economic strata against the higher risk of daily negative emotional symptoms. Other studies have also supported the idea of general self-efficacy as a protective or buffering factor against the negative mental health effects of stressful conditions [21]. This line of reasoning is in accordance with the salutogenic model of health, which highlights the role of various resistance resources in promoting good mental health as they make people more resilient or robust in the face of stressful situations [48–50]. In this regard, general self-efficacy can be viewed as a cognitive resistance resource that may contribute to the reduction of social disparities in mental health among adolescents.

Furthermore, research has repeatedly revealed poorer self-reported mental health [2, 4, 11, 20, 21, 51] and lower general self-efficacy (e.g., [21, 27, 52, 53]) among adolescent girls than boys. One study found that the observed inverse relationship between general self-efficacy and depressive symptoms was stronger for girls than for boys [11]. This may be related to gender role socialization processes [54], which possibly result in girls having less confidence in their ability to handle difficult situations [21, 27] while simultaneously placing more importance on being accomplished [55]. Consequently, GSE beliefs might be more salient for their mental health compared to boys’. Such findings point to the need for a gendered perspective in research addressing these issues. The few studies that have investigated the mediating and moderating role of general self-efficacy in the relationship between family socio-economic position and mental health have not addressed gender differences. Yet such knowledge on gender differences would increase our understanding of antecedents of mental health in adolescent population subgroups and contribute to further development of theory, and to the evidence base for better targeted interventions.

Additionally, the majority of studies investigating general self-efficacy as an antecedent of mental health outcomes have employed relatively small samples. Therefore, there is a need to examine these relationships in larger samples of the general adolescent population.

Finally, research in this area has predominantly focused on negative mental health outcomes [15, 56]. However, it is possible for adolescents to simultaneously have high or low scores on both negative and positive measures of mental health, supporting the proposition that such measures capture (to some degree) different aspects of mental health and should thus be regarded as separate outcomes in research [56]. The current study focuses on subjective well-being, thereby helping answer the call for more research on the antecedents of positive mental health [56, 57].

### Aim and hypotheses

Based on the reviewed literature, the main aims of this study were: (1) to investigate the role of general self-efficacy as a mediator and a moderator of the effect of family SES on subjective well-being in a large population-based Norwegian adolescent sample, and (2) to assess whether there are gender differences in the aforementioned mediated and moderated effects. The following hypotheses were formulated based on existing research and theory:

- H1: Family SES is positively associated with general self-efficacy, which is positively associated with subjective well-being, such that family SES has an indirect positive effect on subjective well-being through general self-efficacy.
- H2: The mediated effect in H1 is significantly greater among adolescent girls than boys.
- H3: Family SES is positively associated with subjective well-being (i.e., lower family SES is associated with lower well-being). This association is smaller among adolescents with higher general self-efficacy than among those with lower general self-efficacy.
- H4: The moderated effect in H3 additionally varies by gender. This hypothesis was exploratory in nature, as previous research did not suggest the direction of any potential interaction effect.

## Methods

### Study design

This study draws on cross-sectional data from the national population-based public health survey program among Norwegian adolescents, “Ungdata”. The program is coordinated by the Norwegian Social Research Institute [58], whereas the regional Drug and Alcohol Competence Centers [59] are responsible for local data collection at the counties that usually take place triennially.

### Data collection and study sample

Data for the current study stems from the 2021 data collection in Vestfold and Telemark county. All secondary school students in the county`s 23 municipalities were invited to take part. The questionnaire was administered by the schools and answered by students online during school hours [60]. In total, 22,028 students responded to the questionnaire, reflecting a response rate of 86% in lower secondary schools and 72% in upper secondary schools [61].

The analytic sample in this study includes 17,941 respondents with non-missing values on the study measures included in the multi-variable analyses. Attrition analyses revealed that the included students reported somewhat higher subjective well-being, family SES, and general self-efficacy than those who were excluded due to missing data. Additionally, those included in the sample were slightly older and consisted of a higher proportion of girls (see Table 1).

**Table 1:** Attrition analysis for the study

	Not included in study sample			Included in study sample				
				*n* = 17,941				
Variable	n	M	SD	M	SD	M diff	95% CI M diff	
Subjective well-being	3651	6.81	2.32	7.11	1.91	-.29	-.36	-.22
Family SES	3256	4.11	0.96	4.20	0.89	-.09	-.12	-.06
General self-efficacy	1085	2.80	0.69	2.93	0.62	-.13	-.17	-.09
Age	3428	16.16	1.62	16.31	1.61	-.15	-.21	-.09
		n	%	n	%	Chi-square results		
Gender								
*Boys*		2077	57.1	8699	48.5			
*Girls*		1562	42.9	9242	51.5	X^2^(1,21,580) = 89.28, *p* <.001		

Values for those not included in the study are based on those who had filled in the questionnaire on actual variables. Range Subjective well-being = 0–10, Family SES = 1–5, General self-efficacy = 1–4

*M* Mean, *SD* Standard deviation, *M* diff Difference between means, *CI* Confidence interval

### Ethics

The current study used pre-existing anonymous data. Participation in the study was voluntary and based on informed consent. This included passive consent from parents/guardians of participants younger than 18 years, i.e., if they did not wish for their children to participate, they took action to communicate this, otherwise it was assumed that they consented [62]. The study was approved by the Norwegian Center for Research Data (Project title: Ungdata 2020–2022, project number 821474). The Norwegian Social Research Institute provided access to the data for this research project.

### Measurements

#### Subjective well-being

Based on previous recommendations [56], we assessed the dependent variable of the study, subjective well-being, through one item capturing adolescents` overall appraisal of life satisfaction. This was measured by a variant of the Cantril`s ladder scale: “Generally speaking, where do you stand on this scale these days?” The scale ranged from 0 (worst possible life) to 10 (best possible life). Single-item measures of subjective well-being are widely used in research, especially in large-scale studies with limited questionnaire space such as World Values Survey and the Gallup World Poll [63], and have shown good validity and reliability [64, 65].

#### Family SES

As research indicates that subjective measures of SES can be an important predictor of health [66], we included a subjective measure of adolescents` family SES as the independent variable in the study. This was assessed through the item: “Financially, has your family been well off, or badly off, over the past two years?” The response categories were as follows (reverse coded): We have been badly off the whole time = 1, We have mostly been badly off = 2, We have neither been well off nor badly off = 3, We have mostly been well off = 4, We have been well off the whole time = 5. This variable was treated as a continuous measure.

#### General self-efficacy

The mediator and main moderator variable in this study, general self-efficacy, was assessed by the Norwegian short version of the General Perceived Self-Efficacy Scale [67, 68] and included the following five statements: “I always manage to solve difficult problems if I try hard enough”, “I feel confident that I would be able to deal with unexpected events in an effective way”, “I remain calm when I face difficulties because I trust my ability to cope”, “If someone opposes me, I can find the means and ways to get what I want”, “If I'm in a predicament, I usually find a way out”. Response categories were: Completely wrong = 1, Quite wrong = 2, Quite true = 3, and Completely true = 4. A mean score was calculated for respondents who had answered at least three of the five items. The final variable ranged from 1 to 4, with higher scores indicating stronger general self-efficacy. Both the original ten-item scale and the Norwegian short scale version have previously shown good psychometric properties [17, 53, 69–71]. Internal consistency in this study`s sample was also good (α = 0.87).

#### Gender and age

Gender was measured with response options “Boy” (= 0) and “Girl” (= 1). Class level was used as a proxy for age (range = 14 to 19 or respectively first year of lower secondary school to third year of upper secondary school).

### Data analyses

**Fig. 1:**
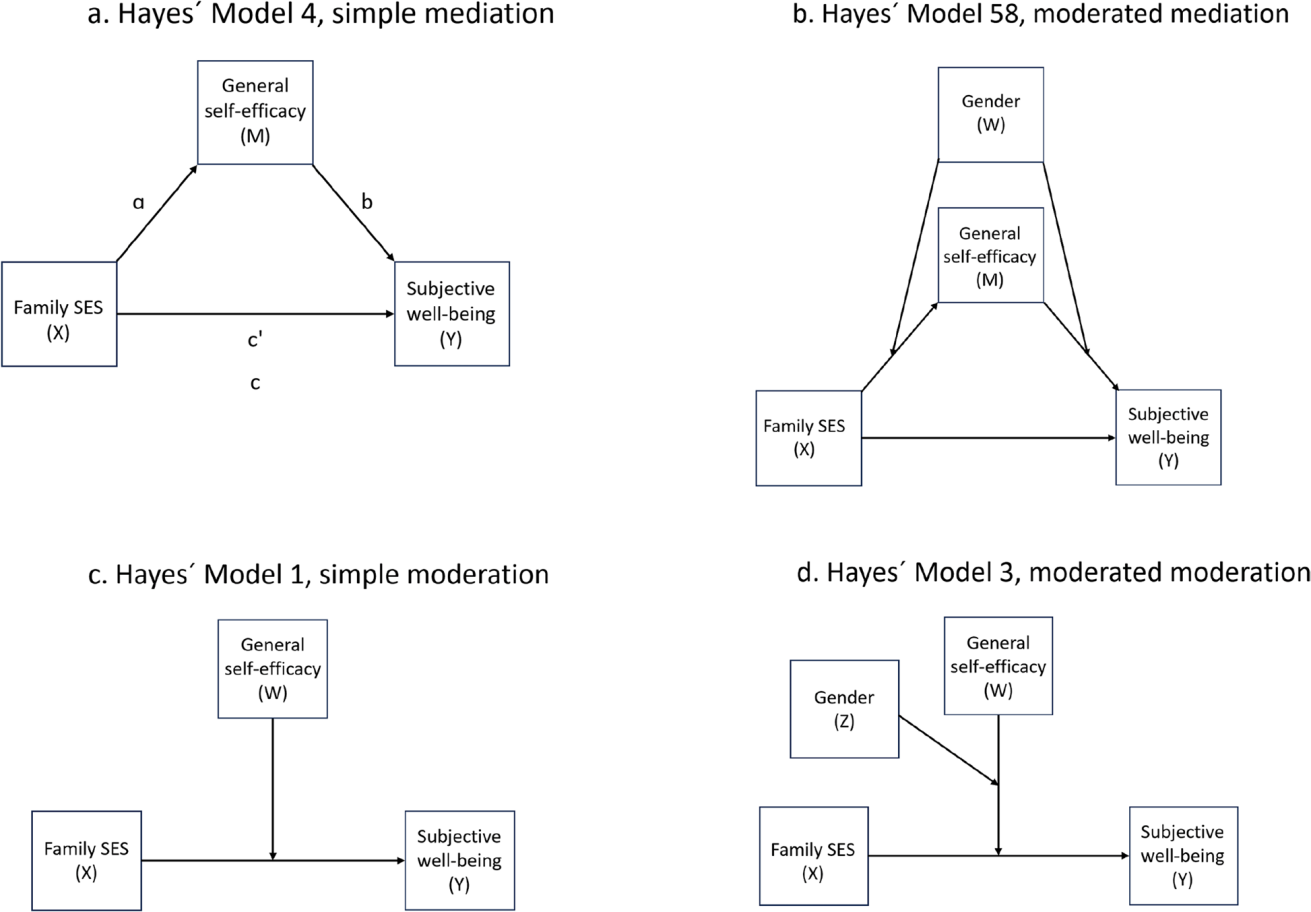
**a**-**d**. Tested mediation and moderation models

All data analyses were performed with IBM SPSS 29. Means and standard deviations of included variables, and correlations between variables, were computed for the total sample and for boys and girls separately. We used the PROCESS application in SPSS 29 [72] to test our hypotheses through four models. The *first model* (simple mediation, Hayes` model 4) tested whether the effect of family SES (X) on subjective well-being (Y) was mediated by general self-efficacy (M), controlling for gender and age (see Fig. 1a). The *second model* (moderated mediation, Hayes` model 58) tested whether the mediation effect from the first model was moderated by gender (W), controlling for age (see Fig. 1b). The *third model* (simple moderation, Hayes` model 1) tested whether family SES (X) and general self-efficacy (W) interacted in their effect on subjective well-being (Y), controlling for gender and age (see Fig. 1c). The *fourth model* (moderated moderation/three-way interaction, Hayes` model 3) tested whether the interaction effect in model 3 was moderated by gender (Z), controlling for age (see Fig. 1d). The PROCESS application provides standardized regression coefficients only in plain mediation models. Thus, these were assessed along with the unstandardized ones only in the first/simple mediation model. Statistical significance of test results was established if the 95% confidence interval estimates did not include zero [72]. Test of indirect effects were based on bootstrapped confidence intervals with 5000 bootstrap samples. Simple slopes analyses were conducted to aid in the interpretation of significant interaction effects. Assumptions for multi-variable linear regression analysis (non-collinearity, normality, homoscedasticity, and linearity) were tested and found to be met [73].

To address the potential bias associated with the systematic sample attrition, we also conducted sensitivity analyses of the four models using an imputed dataset generated with the Expectation–Maximization algorithm (missing data for gender were not imputed, as this method does not support imputation of categorical variables).

## Results

### Descriptive and correlation analyses

Table 1 showed that there were slightly more girls (51.5%) than boys (48.5%) in the sample. Further descriptive and correlation results for the total sample, and for boys and girls separately, are presented in Table 2. Mean age was 16.31 years. In general, the sample reported relatively high subjective well-being, family SES, and general self-efficacy, although boys reported higher subjective well-being and general self-efficacy than girls (correlation [*r*] between gender and: subjective well-being = −0.21, general self-efficacy = −0.22). For both boys and girls, subjective well-being had a small to moderate-sized positive correlation with family SES (*r* = 0.27). Family SES correlated positively (and similarly across genders) with general self-efficacy (*r* = 0.18). The highest correlation for the total sample was found between general self-efficacy and subjective well-being (*r* = 0.35); however, this correlation was higher for girls than for boys (*r* = 0.38 and 0.25, respectively).

**Table 2:** Descriptive statistics and correlations between variables included in the analyses

	Variables	Range	M	SD	1	2	3	4
Total sample (*N* = 17,941)	1. Subjective well-being	0–10	7.11	1.91				
	2. Family SES	1–5	4.20	0.89	.28			
	3. General self-efficacy	1–4	2.93	0.62	.35	.19		
	4. Gender	─	─	─	-.21	-.05	-.22	
	5. Age	14–19	16.31	1.61	-.04	-.02	.07	.04
Boys (*n* = 8699)	1. Subjective well-being	0–10	7.53	1.78				
	2. Family SES	1–5	4.25	0.87	.27			
	3. General self-efficacy	1–4	3.07	0.61	.25	.18		
	5. Age	14–19	16.25	1.58	-.10	-.04	.08	
Girls (*n* = 9242)	1. Subjective well-being	0–10	6.71	1.96				
	2. Family SES	1–5	4.15	0.91	.27			
	3. General self-efficacy	1–4	2.80	0.59	.38	.18		
	5. Age	14–19	16.37	1.64	.01	-.01	.07	

Boys = 0, Girls = 1. Higher scores on continuous variables reflect higher levels of the given indicator. Correlations with Gender are based on Point biserial correlation analyses, correlations between Family SES and continuous variables are based on Spearman correlation analyses, otherwise Pearson correlations are presented

*M* Mean, *SD* Standard deviation

### Simple and moderated mediation analyses

#### First model

We first conducted a simple mediation analysis to assess whether general self-efficacy mediated the effect of family SES on subjective well-being (see Fig. 1a). After controlling for gender and age, we found that family SES positively predicted the adolescents` subjective well-being (total effect path (c), *B* = 0.61, 95% *CI* = 0.58–0.64) and their general self-efficacy (path ɑ, *B* = 0.12, 95% *CI* = 0.11–0.13); and general self-efficacy positively predicted their subjective well-being (path b, *B* = 0.88, 95% *CI* = 0.83–0.92) (see Table 3). Most of the total effect of family SES on subjective well-being was direct (direct path (c'), *B* = 0.50, 95% *CI* = 0.48–0.53), while a small part was channeled indirectly through general self-efficacy (indirect effect, ɑ*b, *B* = 0.11, 95% bootstrapped *CI* = 0.10–0.12). These results provided support for hypothesis 1.

**Table 3:** Simple mediation analysis of the effect of family SES on subjective well-being (*n* = 17,941)

Path	B	B SE	β	t	p	95% CI for B	
						Lower	Upper
Family SES → GSE (ɑ)	0.12	0.01	0.18	24.47	<.001	0.11	0.13
GSE → SWB (b)	0.88	0.02	0.28	40.36	<.001	0.83	0.92
Total path (c)	0.61	0.02	0.28	40.54	<.001	0.58	0.64
Direct path (c')	0.50	0.02	0.23	34.39	<.001	0.48	0.53
Indirect path	0.11	0.01	0.05	─	─	0.10	0.12

The model is controlled for gender and age

Range Family SES = 1–5, GSE = 1–4, SWB = 0–10. Based on Hayes´ PROCESS model 4

*B* Unstandardized regression coefficient, *B SE* Standard error of B, *β* Standardized regression coefficient, *CI* Confidence interval, *SES* Socio-economic status, *GSE* General self-efficacy, *SWB* Subjective well-being. Inference result for the indirect path is bootstrapped (*N* = 5000)

#### Second model

**Fig. 2:**
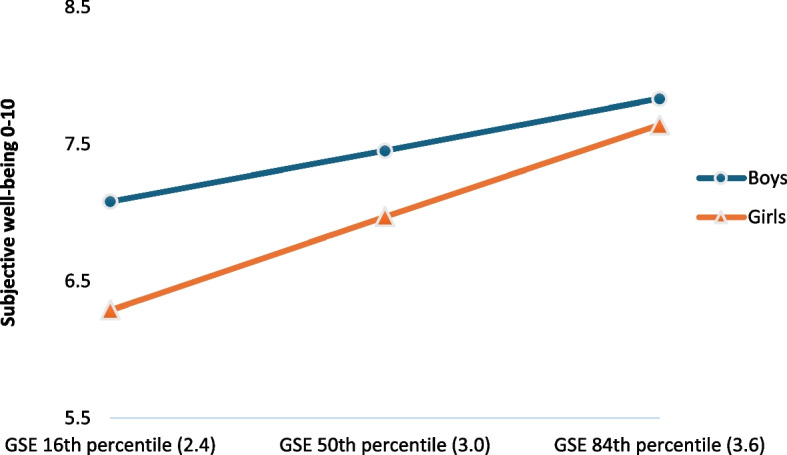
Interaction between General self-efficacy and Gender on Subjective well-being. Note: GSE = General self-efficacy. Range GSE = 1–4

To assess whether the mediated effect (by general self-efficacy) of family SES on subjective well-being was moderated by gender, we conducted a moderated mediation analysis, allowing gender to moderate paths ɑ and b while controlling for age (see Fig. 1b and Table 4). Results revealed a significant interaction effect only for path b (*B* = 0.50, 95% *CI* = 0.42–0.58), showing a larger predictive effect of general self-efficacy on subjective well-being for girls than for boys (*B* = 1.13, 95% *CI* = 1.07–1.19 and *B* = 0.63, 95% *CI* = 0.57–0.69 respectively). The simple slopes figure (Fig. 2) of this interaction effect shows that for both genders, subjective well-being increases with higher general self-efficacy, but more so for girls than boys (i.e., the line is steeper for girls than boys). At a low level of general self-efficacy (16th percentile), the gender gap in subjective well-being is largest, with girls reporting lower subjective well-being than boys. The gender gap diminishes at moderate general self-efficacy (50th percentile): girls catch up a bit with boys on subjective well-being scores. The gender gap is smallest at high levels of general self-efficacy.

**Table 4:** Moderated mediation model by gender (paths ɑ and b) (*n* = 17,941)

Path	B	B SE	t	p	95% CI for B	
					Lower	Upper
Family SES x Gender → GSE (ɑ)	0.00	0.01	−0,22	0.826	−0.02	0.02
GSE x Gender → SWB (b)	0.50	0.04	11.76	<.001	0.42	0.58
GSE → SWB Boys (b)	0.63	0.03	20.71	<.001	0.57	0.69
GSE → SWB Girls (b)	1.13	0.03	37.14	<.001	1.07	1.19
Indirect path Boys	0.08	0.01	─	─	0.06	0.09
Indirect path Girls	0.14	0.01	─	─	0.12	0.15
Index of moderated mediation	0.06	0.01	─	─	0.04	0.08

The model is controlled for age

Range Family SES = 1–5, GSE = 1–4, SWB = 0–10. Based on Hayes´ PROCESS model 58

*B* Unstandardized regression coefficient, *B SE* Standard error of B, *CI* Confidence interval, *SES* Socio-economic status, *GSE* General self-efficacy, *SWB* Subjective well-being. Inference results for the indirect path are bootstrapped (*N* = 5000)

Due to at least one of paths ɑ and b being moderated by gender, the indirect effect of family SES on subjective well-being through general self-efficacy was also significantly moderated by gender, showing a larger indirect effect for girls than for boys (*B* = 0.14, 95% bootstrapped *CI* = 0.12–0.15 and *B* = 0.08, 95% bootstrapped *CI* = 0.06–0.09 respectively; index of moderated mediation = 0.06, 95% bootstrapped *CI* = 0.04–0.08). These results supported the expectation outlined in hypothesis 2.

### Simple and moderated moderation analyses

#### Third model

**Fig. 3:**
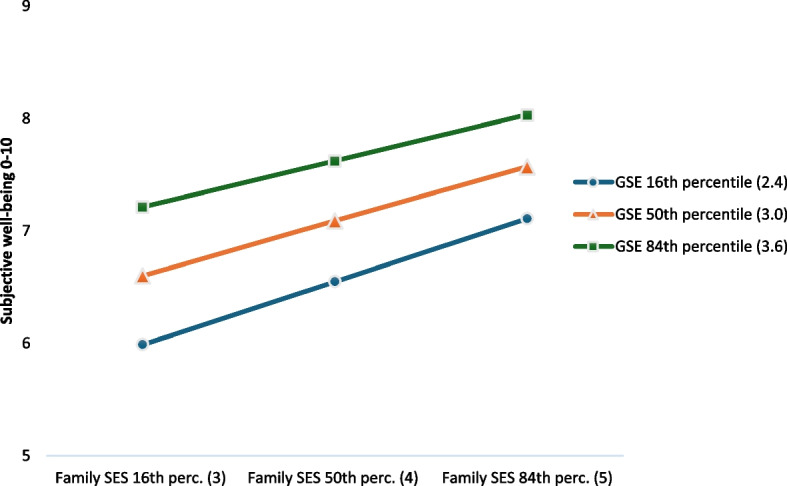
Interaction between Family SES and General self-efficacy in their effect on Subjective well-being. Note: SES = Socio-economic status; GSE = General self-efficacy. Range Family SES = 1–5, GSE = 1–4. Based on Hayes´ PROCESS model 58 (simple moderation)

To assess whether general self-efficacy also could act as a moderator of the effect of family SES on subjective well-being, we first conducted a simple moderation analysis where we included family SES as the focal predictor in the model, subjective well-being as the dependent variable, and general self-efficacy as the moderator, controlling for age and gender (see Fig. 1c). Results revealed a small but significant interaction effect between family SES and general self-efficacy in their effect on subjective well-being (*B* = −0.13, 95% *CI* = −0.17,−0.09) (see Table 5). The Johnson-Neyman method showed that the positive effect of family SES on subjective well-being was statistically significant at all values within the observed range of general self-efficacy, but that the effect was slightly and significantly decreasing with increasing levels of general self-efficacy (effect at different values of general self-efficacy: 2.4/16th percentile (*B* = 0.56, 95% *CI* = 0.53–0.60), 3.0/50th percentile (*B* = 0.48, 95% *CI* = 0.46–0.51), 3.6/84th percentile (*B* = 0.41, 95% *CI* = 0.37–0.45). The simple slope figure for this interaction effect (see Fig. 3) indicates that adolescents who report lower family SES will benefit slightly more (in terms of higher subjective well-being) from increasing their general self-efficacy than adolescents who report higher family SES. Despite small effects, these findings lend support to hypothesis 3.

**Table 5:** Simple moderation analysis of the effect of family SES on subjective well-being (*n* = 17,941)

	B	B SE	t	p	95% CI for B	
					Lower	Upper
R^2^ = 0.201, ∆R^2^ due to interaction = 0.002						
Family SES x GSE → SWB	−0.13	0.02	−6.00	<.001	−0.17	−0.09
Effects of Family SES on subjective well-being at values of GSE						
2.4 (16th percentile)	0.56	0.02	32.15	<.001	0.53	0.60
3.0 (50th percentile)	0.48	0.02	32.28	<.001	0.46	0.51
3.6 (84th percentile)	0.41	0.02	18.76	<.001	0.37	0.45

The model is controlled for gender and age

Range Family SES = 1–5, GSE = 1–4, SWB = 0–10. Based on Hayes´ PROCESS model 1

*B* Unstandardized regression coefficient, *B SE* Standard error of B, *CI* Confidence interval, *SES* Socio-economic status, *GSE* General self-efficacy, *SWB* Subjective well-being

#### Fourth model

To test whether the interaction effect from the simple moderation analysis above was moderated by gender, we introduced gender as a second moderator in the model (see Fig. 1d), controlling for age. Results from this three-way interaction (moderated moderation) analysis revealed no moderation by gender of the moderated effect (by general self-efficacy) of family SES on subjective well-being (family SES*general self-efficacy*gender = *B* = 0.09, 95% *CI* = 0.00–0.17). In the absence of a significant three-way interaction effect, the simpler two-way interaction model where the effect of family SES on subjective well-being varies by general self-efficacy is therefore the preferred model.

#### Additional analyses

To explore the validity of results from these four tested models, we conducted equivalent analyses with a more objective measure of SES (as the focal predictor) available in Ungdata. This SES measure aggregates six items intended to capture family material wealth, parent´s educational level, and family cultural capital [74] (see description in Additional file 1). Results for all four models were identical to those of our main analyses above (see Additional files 2–4), but we found weaker effects of this second SES measure on subjective well-being and general self-efficacy in the simple mediation analysis (Additional file 2), and a weaker interaction effect in the simple moderation analysis (Additional file 4).

We also conducted sensitivity analyses using an imputed dataset. Results of the first three models closely mirrored those of the original analyses (see Additional files 5–7), while the three-way interaction effect in model 4 increased slightly and reached statistical significance, indicating a larger interaction effect between family SES and general self-efficacy for boys than girls (see Additional file 8). Our findings for model 4 from the main/original analyses above, which we focus on and address in the discussion, should therefore be viewed with caution.

## Discussion

This study was aimed at investigating the role of general self-efficacy as a potential mediator and moderator of the effect of family SES on adolescents´ subjective well-being, and whether such effects differed by gender. As hypothesized, we found that some of the effect of the adolescents´ family SES on their well-being was channeled via their general self-efficacy beliefs, and that this indirect effect was stronger for girls than for boys. Furthermore, in line with our hypothesis, we found that general self-efficacy also had a moderating effect on this relationship, i.e. there was a somewhat weaker association between family SES and subjective well-being for those with higher general self-efficacy than for those with lower general self-efficacy. This interaction effect was not moderated by gender.

Although the influence of family´s socio-economic situation on adolescent mental health is well-established [9], knowledge about the mechanisms by which such an effect occurs is still limited [13, 14]. This study contributes to this theoretical knowledge base by showing that parts of the effect of family SES on subjective well-being were mediated by general self-efficacy perceptions. This finding aligns with the two existing studies that already have tested this assumption [28, 39], respectively with health-related quality of life and psychosomatic symptoms as mental health outcomes. The first part of this mediation result, i.e., the socio-economic inequality in general self-efficacy among adolescents, is consistent with theoretical and empirical literature that suggests that adolescents´ family socio-economic conditions influence their general coping expectations through access to direct and vicarious experiences of mastery and supportive and encouraging social environments (e.g., [15, 26, 29, 32]). For instance, research consistently shows that children and adolescents living in socio-economically less advantaged circumstances have lower access to supportive relationships with parents [15], peers and teachers, and lower access to organized leisure activities [9] and good neighborhood facilities for unorganized leisure pursuits [9, 34]. Lack of or inadequate access to the aforementioned can stand in the way of positive experiences that help promote general self-efficacy beliefs. Studies demonstrate, for example, that adolescents´ general self-efficacy is associated with factors in various arenas of their daily lives, including in their homes (e.g., family supportiveness) and neighborhoods (e.g., neighborhood safety) [46], in the school context (e.g., good grades) and among peers (e.g., peer support) [42], and in the physical activity/leisure context (e.g., positive experiences in organized sport) [44, 45].

The second part of the mediation result corresponds with previous research and theory that indicates a positive impact of adolescents´ general perceptions of self-efficacy on their mental health [11, 22] – likely occurring due to the more positive outlook on their ability to overcome challenges in life, a higher dedication and engagement in meaningful activities, and a higher adoption of mental health promoting activities (e.g., [17, 25, 27]). However, to the best of our knowledge, these potential mediating mechanisms for the effect of family SES on general self-efficacy and, further, for the effect of general self-efficacy on mental health, have not been formally tested. Theory in the field would thus benefit from expanding our simple mediation model to a more comprehensive one that simultaneously tests the different suggested pathways of the effect between family SES and mental health, including both serial and parallel mediation paths [72].

Knowledge about the varying determinants of health, and varying health impact of public health efforts, in different population subgroups, is of interest for optimal targeting of public health initiatives [15]. Our findings show that the stronger indirect effect of family SES on subjective well-being among girls than among boys, through general self-efficacy, was not due to differing effects of family SES on general self-efficacy but was attributed to a differing impact of general self-efficacy on subjective well-being. That general self-efficacy had a greater impact on girls´ than boys´ well-being in our study is in line with a previous study addressing depressive symptoms as the outcome [11], in which an inverse association between general self-efficacy beliefs and depressive symptoms was found for both genders, but was significantly stronger among girls. Our results suggest that, while living in a higher SES family is equally beneficial for both girls´ and boys´ general self-efficacy, those general self-efficacy gains (derived from living in a higher SES family) are especially instrumental in promoting girls´ compared to boys´ well-being.

The gender difference in the effect of general self-efficacy on subjective well-being may have several explanations. Boys, compared to girls, consistently report a more robust belief in their ability to navigate challenging tasks and situations (e.g., [21, 27]), presumably attributable to different gender role socialization processes [54]. For boys, having a relatively strong belief in their capacity to manage demanding circumstances may be perceived as more commonplace, which could potentially lessen its impact on their well-being. Conversely, for girls, possessing a higher level of self-efficacy might be perceived as something less common but positive and desirable, which could consequently have a greater influence on their well-being. Another explanation could be that self-efficacy for girls entails more social and sharing experiences than for boys. With personal experiences of mastery being one of the main sources of self-efficacy [29], and girls being more likely to share their successes with friends [75], it could be that the social aspects and benefits of sharing achievements with significant others (e.g., demonstrating competence and establishing warm, supportive relationships) could contribute to the stronger relationship between self-efficacy and well-being for girls compared to boys. Yet another explanation could be that girls, more so than boys, have internalized the characteristics of being accomplished as important (referred to by some as the “good girl syndrome”) [55], and therefore indications of mastery would relate more strongly to girls’ subjective well-being. Despite such explanations, our findings suggest that – due to both lower levels of general self-efficacy, lower well-being, and a larger beneficial effect of heightened general self-efficacy on well-being among girls than among boys – it may be particularly useful to target adolescent girls with general self-efficacy promoting efforts in mental health promotion work.

In line with the concept of *resistance resources* in the salutogenic model of health [48, 76], we also investigated the possibility that general self-efficacy not only acts as a mediator between family SES and subjective well-being, but that it also buffers or protects against the detrimental effects of living in a family with fewer socio-economic resources. This proposition was confirmed in our simple moderation model. Furthermore, the moderated moderation model revealed that this conditional effect of family SES on well-being was independent of gender. It thus appears that, irrespective of gender, higher general self-efficacy is more beneficial for the subjective well-being of adolescents from lower – than those from higher SES families. This finding corresponds with results from a similar study among Danish adolescents [16]; and to studies indicating that general self-efficacy protects against the negative mental health consequences of other stressful life situations [21]. As such, general self-efficacy may represent an important cognitive resistance resource for adolescents.

General self-efficacy, as it is presumably more amenable to change than the socio-economic situation of one´s family, may be especially important to target in interventions that include adolescents from lower SES-families. Such efforts can help reduce socio-economic disparities in mental health in the adolescent population. Previous research points to various arenas (e.g., in the home, in school, among peers, and in leisure activities [46, 47]) where general self-efficacy can be enhanced independent of the family´s socio-economic situation. As previously highlighted, these arenas should focus on providing opportunities for experiences of mastery directly or by observing relevant successful role models, and expose young people to encouraging and supportive significant others [29, 30]. However, although the interaction effect involving family SES and general self-efficacy was statistically significant, its size was very small (∆R^2^ due to interaction = 0.002), which raises questions about its practical significance. Nevertheless, in a public health context, even small effects can be meaningful when applied to large populations [77]. Additional research is necessary to validate the potential protective role of general self-efficacy against the negative impacts of low family SES. Furthermore, the alternative interpretation of the significant interaction effect should be considered – that residing in a high SES-family may help alleviate the adverse effects of low general self-efficacy on well-being.

This study used subjective family SES as the focal predictor in the analyses. The robustness of our findings was confirmed through similar mediation and moderation results in sensitivity analyses applying a more objective, but still self-reported, measure of family SES encompassing material wealth, parental education level, and cultural capital. These additional analyses revealed weaker effects of family SES on general self-efficacy and SWB, and a smaller interaction effect between family SES and general self-efficacy on SWB. The stronger impact of the subjective SES measure aligns with prior research [78, 79]. The measure of perceived household financial constraints in this study likely reflects adolescents’ personal experiences and emotions regarding their family’s economic situation, which may more directly influence their self-efficacy beliefs and well-being compared to the objective measure. Researchers [80] also emphasize the importance of perceptions of relative socio-economic position, which may be captured by subjective measures rather than objective ones. Such social comparison is thought to be closely linked to psychological processes likely relevant for the development of general self-efficacy and SWB among adolescents. Therefore, using subjective SES measures alongside objective ones is considered essential for research on SES impacts on adolescent mental health [80].

### Strengths and limitations of the study

This study, which is based on data from a large sample of the general adolescent population in a Norwegian county, adds important knowledge to the evidence base on pathways by which the socio-economic situation of adolescents` families affects their well-being; and of factors that can contribute to the reduction of socio-economic and gender-based disparities in adolescents´ positive mental health. However, several limitations should be taken into account when interpreting the results. Given the cross-sectional design of the study, we cannot conclude about the directions of causality in the observed associations. For example, it is likely that one´s general perceived ability to tackle challenging situations is affected by one´s perceptions of well-being. Also, the analyses controlled for gender and age, but did not include other potential confounding variables, such as ethnicity and family structure, which were unavailable in the Ungdata dataset. Although available variables like parental engagement and social support were initially considered as covariates, they were ultimately excluded as the literature indicates that these factors act as important mechanisms for a positive impact of higher SES on general self-efficacy, and consequently SWB. Thus, including them as controls might likely have led to overadjustment bias [81, 82]. Nonetheless, the lack of controls for all relevant confounders may still have overestimated mediation and moderation results. The study’s findings should therefore be interpreted with caution.

Further, attrition analyses revealed significantly more positive scores on well-being, SES, and general self-efficacy among respondents included in the study compared to those excluded due to missing data. This non-random missingness, resulting from the use of listwise deletion, may introduce some uncertainty into the regression estimates. However, sensitivity analyses conducted with an imputed dataset generally supported the main findings. Nonetheless, the slight change observed in the results for model 4 suggests that this model’s main/original findings should be interpreted with caution and considered in light of the results from the sensitivity analysis.

Finally, generalizability in time might also have been influenced: the data was collected during the Covid-19 pandemic, a period in which adolescents` family SES, well-being, and general self-efficacy might have been affected by Covid-related factors such as layoffs and society closures/restricted movement – and the attendant reductions in social participation and engagement in beneficial activities.

## Conclusion

This study revealed that adolescents´ general sense of self-efficacy plays a dual role in the relationship between the socio-economic situation of their family and their subjective well-being. First, it partly mediates the positive effect of family SES on well-being, and more so among girls than boys due to a larger beneficial effect of general self-efficacy on well-being among girls. Additionally, in another set of analyses, our study revealed that general self-efficacy also moderates the effect of family SES on well-being by protecting against reduced well-being among adolescents living in families with fewer socio-economic resources. Thus, from a public mental health perspective with particular focus on reducing inequalities in subjective well-being among adolescents: besides a continued focus on increasing the socio-economic resources of lower SES-families (as this should improve well-being through various pathways, including via a strengthened general self-efficacy), it appears especially fruitful to support the formation and strengthening of general self-efficacy beliefs among girls and among those living in lower SES-families. Still, the knowledge base in this area is limited and would benefit from more large-scale studies testing more comprehensive models, preferably with longitudinal and experimental designs.

## Supplementary Information

Additional file 1. Description of the aggregated, more objective family SES measure used in additional analyses.

Additional file 2. Results from the simple mediation analysis with objective family SES as the focal predictor.

Additional file 3. Results from the moderated mediation analysis with objective family SES as the focal predictor.

Additional file 4. Results from the simple and moderated moderation analyses with objective family SES as the focal predictor.

Additional file 5. Results from the simple mediation analysis based on the imputed dataset.

Additional file 6. Results from the moderated mediation analysis based on the imputed dataset.

Additional file 7. Results from the simple moderation analysis based on the imputed dataset.

Additional file 8. Results from the moderated moderation analysis based on the imputed dataset.

## Data Availability

The data that support the findings of this study are available for researchers on request to the Norwegian agency for shared services in education and research (https://sikt.no/en/home Accessed 28 June 2024).

## Abbreviation

SES: Socio-economic status
