# Supplementary material for: Effect of family socio-economic status on subjective well-being among Norwegian adolescents: Mediation and moderation effects by general self-efficacy from a gendered perspective
Source: BMC Public Health. 2025 Oct 8;25:3380. doi: 10.1186/s12889-025-24697-7 (PMC12505702; doi:10.1186/s12889-025-24697-7)
Supplement: Supplementary file 1 — Additional file 1. Description of the aggregated, more objective family SES measure used in additional analyses. [file 12889_2025_24697_MOESM1_ESM.docx]

**Description of the aggregated, more objective family SES measure:**

Objective family SES was assessed by combining answers to: (a) four questions from the Family Affluence Scale II (FAS II) (1): 1. “Does your family have a car?” (Response categories: No/Yes, one/Yes, two or more); 2. “Do you have your own bedroom?” (Response categories: Yes/No); 3. “How many times have you travelled somewhere on holiday with your family over the past year?” (Response categories: Never/Once/Twice/More than twice); 4. “How many computers or tablet computers does your family have?” (Response categories: None/One/Two/More than two), (b) one question about the parents´ educational level (“Did your father and mother go to university or to a university college?” (Response categories: Yes/No for each parent)), and (c) one question about the number of books in the home (“How many books do you think there are in your home?” (Response categories: No books/Fewer than 20 books/20 – 100 books/100 – 500 books/500 – 1000 books/More than 1000 books)). In line with instructions given by Bakken et al. (2), response options for each of these 6 questions were coded to represent a scale ranging from 0 (low SES) to 3 (high SES). For example, the parental educational scale included: None of the parents have higher education= 0, One of the parents = 1,5, Both parents = 3. Then a mean score for the four FAS II questions was computed to create one FAS II variable, and finally, a mean objective family SES score was computed from scores on FAS II, parents` educational level, and books in the home for those respondents who had values on at least two of these three dimensions. This aggregated socio-economic status measure has shown good validity (2).

1. Currie C, Molcho M, Boyce W, Holstein B, Torsheim T, Richter M. Researching health inequalities in adolescents: the development of the Health Behaviour in School-Aged Children (HBSC) family affluence scale. Social science & medicine. 2008;66(6):1429-36.

2. Bakken A, Frøyland LR, Sletten MA. Sosiale forskjeller i unges liv. Hva sier Ungdata-undersøkelsene? Oslo: NOVA; 2016. Report No.: 3/16.
