# Supplementary material for: Effect of family socio-economic status on subjective well-being among Norwegian adolescents: Mediation and moderation effects by general self-efficacy from a gendered perspective
Source: BMC Public Health. 2025 Oct 8;25:3380. doi: 10.1186/s12889-025-24697-7 (PMC12505702; doi:10.1186/s12889-025-24697-7)
Supplement: Supplementary file 2 — Additional file 2. Results from the simple mediation analysis with objective family SES as the focal predictor. [file 12889_2025_24697_MOESM2_ESM.docx]

| Additional table. Simple mediation analysis - objective family SES (n= 18094). | | | | | | | |
| --- | --- | --- | --- | --- | --- | --- | --- |
| Path | B | B SE | β | t | p | 95% CI for B | |
|  |  |  |  |  |  | Lower | Upper |
| Objective family SES → GSE (ɑ) | 0.13 | 0.01 | 0.11 | 15.72 | <.001 | 0.12 | 0.15 |
| GSE → SWB (b) | 0.98 | 0.02 | 0.32 | 44.66 | <.001 | 0.94 | 1.03 |
| Total path (c) | 0.37 | 0.03 | 0.10 | 14.14 | <.001 | 0.32 | 0.42 |
| Direct path (c') | 0.24 | 0.03 | 0.07 | 9.62 | <.001 | 0.19 | 0.29 |
| Indirect path | 0.13 | 0.01 | 0.04 | ─ | ─ | 0.11 | 0.15 |
| Note: The model is controlled for gender and age. B= Unstandardized regression coefficient; B SE= Standard error of B; β= Standardized regression coefficient, CI= Confidence interval; SES= Socio-economic status; GSE= General self-efficacy; SWB= Subjective well-being. Inference result for the indirect path is bootstrapped (*N*=5000). Range Objective family SES= 0-3, GSE= 1-4, SWB= 0-10. Based on Hayes´ PROCESS model 4. | | | | | | | |
