# Supplementary material for: Effect of family socio-economic status on subjective well-being among Norwegian adolescents: Mediation and moderation effects by general self-efficacy from a gendered perspective
Source: BMC Public Health. 2025 Oct 8;25:3380. doi: 10.1186/s12889-025-24697-7 (PMC12505702; doi:10.1186/s12889-025-24697-7)
Supplement: Supplementary file 3 — Additional file 3. Results from the moderated mediation analysis with objective family SES as the focal predictor. [file 12889_2025_24697_MOESM3_ESM.docx]

| Additional table. Moderated mediation model by gender (paths ɑ and b) (n= 18094). | | | | | | |
| --- | --- | --- | --- | --- | --- | --- |
| Path | B | B SE | t | p | 95% CI for B | |
|  |  |  |  |  | Lower | Upper |
| Objective family SES x Gender → GSE (ɑ) | 0.00 | 0.02 | 0.11 | 0.910 | -0.03 | 0.04 |
| GSE x Gender → SWB (b) | 0.51 | 0.04 | 11.67 | <.001 | 0.42 | 0.59 |
|  |  |  |  |  |  |  |
| GSE → SWB Boys (b) | 0.73 | 0.03 | 23.60 | <.001 | 0.67 | 0.79 |
| GSE → SWB Girls (b) | 1.24 | 0.03 | 40.08 | <.001 | 1.18 | 1.30 |
|  |  |  |  |  |  |  |
| Indirect path Boys | 0.10 | 0.01 | ─ | ─ | 0.08 | 0.12 |
| Indirect path Girls | 0.16 | 0.02 | ─ | ─ | 0.13 | 0.20 |
| Index of moderated mediation | 0.07 | 0.02 | ─ | ─ | 0.03 | 0.11 |
| Note: The model is controlled for age. B= Unstandardized regression coefficient; B SE= Standard error of B; CI= Confidence interval; SES= Socio-economic status; GSE= General self-efficacy; SWB= Subjective well-being. Inference results for the indirect paths are bootstrapped (*N*=5000). Range Objective family SES= 0-3, GSE= 1-4, SWB= 0-10. Based on Hayes´ PROCESS model 58. | | | | | | |
