# Supplementary material for: Effect of family socio-economic status on subjective well-being among Norwegian adolescents: Mediation and moderation effects by general self-efficacy from a gendered perspective
Source: BMC Public Health. 2025 Oct 8;25:3380. doi: 10.1186/s12889-025-24697-7 (PMC12505702; doi:10.1186/s12889-025-24697-7)
Supplement: Supplementary file 4 — Additional file 4. Results from the simple and moderated moderation analyses with objective family SES as the focal predictor. [file 12889_2025_24697_MOESM4_ESM.docx]

| Additional table. Simple moderation analysis – objective family SES (n= 18094). | | | | | | |
| --- | --- | --- | --- | --- | --- | --- |
|  | B | B SE | t | p | 95% CI for B | |
|  |  |  |  |  | Lower | Upper |
| R² = 0.150, ∆R² due to interaction = 0.0002 | | | | | | |
| Objective family SES x GSE→ SWB | -0.08 | 0.04 | -2.05 | 0.040 | -0.15 | -0.004 |
|  |  |  |  |  |  |  |
| Conditional effects of objective family SES at values of GSE | | | |  |  |  |
| 2.4 (16th percentile) | 0.28 | 0.03 | 8.83 | <.001 | 0.22 | 0.34 |
| 3.0 (50th percentile) | 0.23 | 0.03 | 9.28 | <.001 | 0.19 | 0.28 |
| 3.6 (84th percentile) | 0.19 | 0.04 | 5.19 | <.001 | 0.12 | 0.26 |
| Note: The model is controlled for gender and age. B= Unstandardized regression coefficient; B SE= Standard error of B; CI= Confidence interval; SES= Socio-economic status; GSE= General self-efficacy; SWB= Subjective well-being. Range Objective family SES= 0-3, GSE= 1-4, SWB= 0-10. Based on Hayes´ PROCESS model 1. The three-way interaction analysis based on Hayes´ PROCESS model 3 revealed no moderation by gender of the moderated effect (by general self-efficacy) of objective family SES on subjective well-being (Objective family SES*general self-efficacy*gender = B = 0.11, 95% CI = -0.04;0.26). | | | | | | |
