# Supplementary material for: Effect of family socio-economic status on subjective well-being among Norwegian adolescents: Mediation and moderation effects by general self-efficacy from a gendered perspective
Source: BMC Public Health. 2025 Oct 8;25:3380. doi: 10.1186/s12889-025-24697-7 (PMC12505702; doi:10.1186/s12889-025-24697-7)
Supplement: Supplementary file 5 — Additional file 5. Results from the simple mediation analysis based on the imputed dataset. [file 12889_2025_24697_MOESM5_ESM.docx]

| Additional table. Simple mediation analysis of the effect of family SES on subjective well-being based on the imputed dataset (n=21580). | | | | | | | |
| --- | --- | --- | --- | --- | --- | --- | --- |
| Path | B | B SE | β | t | p | 95% CI for B | |
|  |  |  |  |  |  | Lower | Upper |
| Family SES → GSE (ɑ) | 0.12 | 0.00 | 0.19 | 28.51 | <.001 | 0.12 | 0.13 |
| GSE → SWB (b) | 1.01 | 0.02 | 0.31 | 48.56 | <.001 | 0.97 | 1.05 |
| Total path (c) | 0.63 | 0.01 | 0.29 | 45.22 | <.001 | 0.61 | 0.66 |
| Direct path (c') | 0.51 | 0.01 | 0.23 | 37.50 | <.001 | 0.48 | 0.54 |
| Indirect path | 0.13 | 0.01 | 0.06 | ─ | ─ | 0.11 | 0.14 |
| Note: The model is controlled for gender and age. B= Unstandardized regression coefficient; B SE= Standard error of B; β= Standardized regression coefficient, CI= Confidence interval; SES= Socio-economic status; GSE= General self-efficacy; SWB= Subjective well-being. Inference result for the indirect path is bootstrapped (*N*=5000). Range Family SES= 1-5, GSE= 1-4, SWB= 0-10. Based on Hayes´ PROCESS model 4. | | | | | | | |
