# Supplementary material for: Effect of family socio-economic status on subjective well-being among Norwegian adolescents: Mediation and moderation effects by general self-efficacy from a gendered perspective
Source: BMC Public Health. 2025 Oct 8;25:3380. doi: 10.1186/s12889-025-24697-7 (PMC12505702; doi:10.1186/s12889-025-24697-7)
Supplement: Supplementary file 6 — Additional file 6. Results from the moderated mediation analysis based on the imputed dataset. [file 12889_2025_24697_MOESM6_ESM.docx]

| Additional table. Moderated mediation model by gender (paths ɑ and b), based on the imputed dataset (n= 21580). | | | | | | |
| --- | --- | --- | --- | --- | --- | --- |
| Path | B | B SE | t | p | 95% CI for B | |
|  |  |  |  |  | Lower | Upper |
| Family SES x Gender → GSE (ɑ) | 0.00 | 0.01 | -0.45 | 0.656 | -0.02 | 0.01 |
| GSE x Gender → SWB (b) | 0.45 | 0.04 | 11.12 | <.001 | 0.37 | 0.53 |
|  |  |  |  |  |  |  |
| GSE → SWB Boys (b) | 0.79 | 0.03 | 27.20 | <.001 | 0.73 | 0.85 |
| GSE → SWB Girls (b) | 1.24 | 0.03 | 42.48 | <.001 | 1.18 | 1.30 |
|  |  |  |  |  |  |  |
| Indirect path Boys | 0.10 | 0.01 | ─ | ─ | 0.09 | 0.12 |
| Indirect path Girls | 0.15 | 0.01 | ─ | ─ | 0.13 | 0.17 |
| Index of moderated mediation | 0.05 | 0.01 | ─ | ─ | 0.03 | 0.08 |
| Note: The model is controlled for age. B= Unstandardized regression coefficient; B SE= Standard error of B; CI= Confidence interval; SES= Socio-economic status; GSE= General self-efficacy; SWB= Subjective well-being. Inference results for the indirect path are bootstrapped (*N*=5000). Range Family SES= 1-5, GSE= 1-4, SWB= 0-10. Based on Hayes´ PROCESS model 58. | | | | | | |
