# Supplementary material for: Effect of family socio-economic status on subjective well-being among Norwegian adolescents: Mediation and moderation effects by general self-efficacy from a gendered perspective
Source: BMC Public Health. 2025 Oct 8;25:3380. doi: 10.1186/s12889-025-24697-7 (PMC12505702; doi:10.1186/s12889-025-24697-7)
Supplement: Supplementary file 7 — Additional file 7. Results from the simple moderation analysis based on the imputed dataset. [file 12889_2025_24697_MOESM7_ESM.docx]

| Additional table. Simple moderation analysis of the effect of family SES on subjective well-being, based on the imputed dataset (n= 21580). | | | | | | |
| --- | --- | --- | --- | --- | --- | --- |
|  | B | B SE | t | p | 95% CI for B | |
|  |  |  |  |  | Lower | Upper |
| R² = 0.22, ∆R² due to interaction = 0.002 | | | | | | |
| Family SES x GSE→ SWB | -0.16 | 0.02 | -7.73 | <.001 | -0.20 | -0.12 |
|  |  |  |  |  |  |  |
| Effects of Family SES on subjective well-being at values of GSE | | | | | | |
| 2.4 (16th percentile) | 0.58 | 0.02 | 35.67 | <.001 | 0.55 | 0.61 |
| 3.0 (50th percentile) | 0.48 | 0.01 | 34.43 | <.001 | 0.45 | 0.51 |
| 3.4 (84th percentile) | 0.42 | 0.02 | 23.35 | <.001 | 0.38 | 0.45 |
| Note: The model is controlled for gender and age. B= Unstandardized regression coefficient; B SE= Standard error of B; CI= Confidence interval; SES= Socio-economic status; GSE= General self-efficacy; SWB= Subjective well-being. Range Family SES= 1-5, GSE= 1-4, SWB= 0-10. Based on Hayes´ PROCESS model 1. | | | | | | |
