# Supplementary material for: Effect of family socio-economic status on subjective well-being among Norwegian adolescents: Mediation and moderation effects by general self-efficacy from a gendered perspective
Source: BMC Public Health. 2025 Oct 8;25:3380. doi: 10.1186/s12889-025-24697-7 (PMC12505702; doi:10.1186/s12889-025-24697-7)
Supplement: Supplementary file 8 — Additional file 8. Results from the moderated moderation analysis based on the imputed dataset. [file 12889_2025_24697_MOESM8_ESM.docx]

| Additional table. Moderated moderation analysis of the effect of family SES on subjective well-being, based on the imputed dataset (n= 21580). | | | | | | |
| --- | --- | --- | --- | --- | --- | --- |
|  | B | B SE | t/F | p | 95% CI for B | |
|  |  |  |  |  | Lower | Upper |
| R² = 0.22, ∆R² due to interaction = 0.0003 | | | | | | |
| Family SES x GSE X Gender → SWB | 0.13 | 0.04 | 3.08 | .002 | 0.05 | 0.21 |
|  |  |  |  |  |  |  |
| Interaction effect between Family SES and GSE on SWB for boys and girls | | | | | | |
| Boys | -0.21 | ─ | 53.61 | <.001 | ─ | ─ |
| Girls | -0.09 | ─ | 8.18 | .004 | ─ | ─ |
| Note: The model is controlled for age. B= Unstandardized regression coefficient; B SE= Standard error of B; CI= Confidence interval; SES= Socio-economic status; GSE= General self-efficacy; SWB= Subjective well-being. Range Family SES= 1-5, GSE= 1-4, SWB= 0-10. Based on Hayes´ PROCESS model 3. | | | | | | |
